# Author Response to Comment on: Dynamic Adsorption of Sulfamethoxazole from Aqueous Solution by Lignite Activated Coke

**DOI:** 10.3390/ma14040868

**Published:** 2021-02-11

**Authors:** Haiyan Li, Juan He, Kaiyu Chen, Zhou Shi, Mengnan Li, Pengpeng Guo, Liyuan Wu

**Affiliations:** 1 Beijing Engineering Research Center of Sustainable Urban Sewage System Construction and Risk Control, Beijing University of Civil Engineering and Architecture,100044 Beijing, China; Lihaiyan@bucea.edu.cn (H.L.); louyounan@163.com (J.H.); Chenkaiyuxiao@163.com (K.C.); shizhou2016@163.com (Z.S.); lmn970109@163.com (M.L.); gzpjustice@163.com (P.G.); 2 Beijing Advanced Innovation Center for Future Urban Design, Beijing 100044, China

**Keywords:** Bohart–Adams model, Yoon–Nelson model, logistic model

## Abstract

The authors would like to thank the comment by Diego M. Juela for contributing to helpful scientific discussions and correction suggestions, and to also express thanks to the editors.

## 1. Introduction 

The authors would like to thank the comment by Diego M. Juela for contributing to helpful scientific discussion and correction suggestions [1], and to also express thanks to the editors. The corresponding reply on the comments are listed as the following aspects. 

## 2. Reply

### 2.1. The Bohart–Adams Model

For the first comment point: “the exponential function of the Bohart-Adams model”, indeed, we need to rectify the sign in “Equation (2) of original paper” which should be “−“ instead of “+”, as corrected below.

Y = exp (kt − b)(1)

To be complemented, the practical modeling calculations we performed with Equation (2) were in the form of “Y = exp(kt − b)” in the original paper, the described modelling results in Table 2 was conducted with the right function format, but the editing and inputting errors were regretful, and we will correct these points. Furthermore, some expressions and typing errors mentioned by Diego M. Juela will be updated in a correction paper. 

### 2.2. The Bohart–Adams, Yoon–Nelson, and Thomas Model

The value of the references related to the logistic model [2], as well as the suggestions of Diego M. Juela about the application of the logistic model during fittings are important. However, considering that there were some debating voices concerning this logistic model during the review stage and after the acceptance of our paper [3,4], whether the normalized logistic models or the used exponential functions were the best fitting models needs to be professionally discussed. We believe that this would be a meaningful scientific problem worthy of further study. Nevertheless, we are thankful for the important comment of Diego M. Juela.

## Reference 

## Data Availability

Data is contained within the article.

## References

[1] Juela D.M. (2021). Comments on “Dynamic Adsorption of Sulfamethoxazole from Aqueous Solution by Lignite Activated Coke”. Materials.

[2] Chu K.H. (2020). Breakthrough curve analysis by simplistic models of fixed bed adsorption: In defense of the century-old Bohart-Adams model. Chem. Eng. J..

[3] Hu Q.L., Zhang. Z.Y. (2020). Comment on “Breakthrough curve analysis by simplistic models of fixed bed adsorption: In defense of the century-old Bohart–Adams model”. Chem. Eng. J..

[4] Chu K.H. (2020). Rebuttal to comment on “Breakthrough curve analysis by simplistic models of fixed bed adsorption: In defense of the century-old Bohart-Adams model” *Chem*. Eng. J..

